# Approaches for the isolation and long-term expansion of pericytes from human and animal tissues

**DOI:** 10.3389/fcvm.2022.1095141

**Published:** 2023-01-10

**Authors:** Valeria Vincenza Alvino, Khaled Abdelsattar Kassem Mohammed, Yue Gu, Paolo Madeddu

**Affiliations:** ^1^Bristol Heart Institute, University of Bristol, Bristol, United Kingdom; ^2^Department of Cardiothoracic Surgery, Faculty of Medicine, Assiut University, Asyut, Egypt

**Keywords:** pericytes, regenerative medicine, methods, isolation and expansion, cardiovascular

## Abstract

Pericytes surround capillaries in every organ of the human body. They are also present around the *vasa vasorum*, the small blood vessels that supply the walls of larger arteries and veins. The clinical interest in pericytes is rapidly growing, with the recognition of their crucial roles in controlling vascular function and possible therapeutic applications in regenerative medicine. Nonetheless, discrepancies in methods used to define, isolate, and expand pericytes are common and may affect reproducibility. Separating pure pericyte preparations from the continuum of perivascular mesenchymal cells is challenging. Moreover, variations in functional behavior and antigenic phenotype in response to environmental stimuli make it difficult to formulate an unequivocal definition of *bona fide* pericytes. Very few attempts were made to develop pericytes as a clinical-grade product. Therefore, this review is devoted to appraising current methodologies’ pros and cons and proposing standardization and harmonization improvements. We highlight the importance of developing upgraded protocols to create therapeutic pericyte products according to the regulatory guidelines for clinical manufacturing. Finally, we describe how integrating RNA-seq techniques with single-cell spatial analysis, and functional assays may help realize the full potential of pericytes in health, disease, and tissue repair.

## 1. Background

Benjamin Rouget was the first to describe and give his name to a particular type of cell within the capillary basement membrane with projections protruding to enwrap the vessel wall (1). In 1923, Zimmerman renamed these Rouget cells “Pericytes.” Under this broad name, they included many forms of cells from different tissue origins located around capillaries (2). Then, research on pericytes entered a hibernation period until 20 years ago, when the pericytes’ regenerative potential started attracting translational interest (3, 4). Nonetheless, a discrepancy persists in the literature regarding pericytes vs. other cardiovascular cells. For instance, pericytes are plentiful in the heart. However, there were only 73 publications on *cardiac pericytes* in PubMed during 2021 (out of 687 publications retrievable using the general term “*pericytes*”) compared with 5,400 for *cardiomyocytes* and 1,150 for *coronary endothelial cells*. This data highlights the need for more research on this cell population.

**FIGURE 1:**
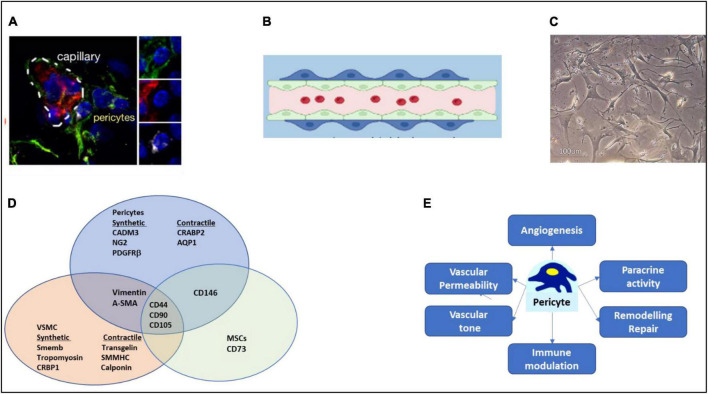
Characteristics of pericytes. **(A)** Immunofluorescence microscopy image of NG2 positive (green) pericytes, with endothelial cells labeled in red and nuclei in blue. **(B)** Cartoon of pericyte around a capillary lined internally by endothelial cells. **(C)** Aspect of pericytes in culture. **(D)** Overlapping of antigens with other mesenchymal cells. For clarity, refer to text for the overlapping with perivascular fibroblasts. **(E)** Key pericyte functions. Image in **(A)** has been reproduced with permission from the original article in Avolio et al. (3).

Several teams have reported technologies for the isolation and derivation of pericytes. Culture expansion allows the generation of stocks for use as a product for cell therapy and tissue engineering. Nonetheless, the consensus on pericyte-related techniques remains far behind that of human endothelial cells (ECs) (5–7). In particular, the diversity of pericytes’ properties and antigenic expression in different organs, together with the overlapping with other perivascular mesenchymal cells, represents a significant obstacle to using one method that fits all experimental conditions. *Ad hoc* standard operating procedures (SOP) may better accomplish distinct scientific/medical goals. For instance, *pure* pericyte products are necessary for obtaining regulatory approval for patients’ treatment. *Semi-purified* preparations are more suitable for deciphering the transition of pericytes across different antigenic and functional phenotypes in response to environmental stimuli. The main characteristics of pericytes are illustrated in Figure 1 and relevant text sections.

## 2. Aims

This review article aims to present, compare, and discuss the experience of deriving pericytes from different organs and species. We initially report current nomenclature, functional roles, and antigenic profiles that distinguish pericytes from other perivascular cell populations. The main body of the review is then devoted to (*i*) documenting the pros and cons of current techniques for isolation, expansion, and characterization of *bona fide* pericytes, (*ii*) suggesting avenues for standardization and harmonization, (*iii*) upgrading protocols for clinical manufacturing, and (*iv*) prospecting the potential of novel single-cell technologies in advancing pericyte research.

## 3. Origin and nomenclature of pericytes

Developmentally, pericytes in the cephalic region and thymus originate from the neuroectoderm. The ones found in the liver, lungs, and heart derive from mesothelium. The mesoderm gives rise to pericytes in other organs (such as kidneys, liver, and pancreas) (8–10). Intriguingly, Yamazaki et al. proposed that a subgroup of pericytes may also derive from the hematopoietic cell lineage (11). Tracing studies may help reveal additional embryological contributors to vascular pericytes.

In the adult organism, microvascular pericytes are classically located within the basement membrane in direct contact with capillary ECs in different organs. In addition, pericytes are present in the vascular adventitia, the blood vessel’s outer layer near the *vasa vasorum* (3, 12). The nomenclature of these intravascular pericytes is inconsistent: the term “*adventitial stromal cells/CD34* + *cells*” refers to the *in situ* expression of CD34, a transmembrane glycoprotein, while “*adventitial pericyte-like progenitors*” denotes their plasticity and positivity for typical stem cell markers (12–15). For clarity, we will refer to these cells as adventitial pericytes.

## 4. Functional roles of pericytes

Pericytes are essential for vasculature’s physiological stability, as exemplified by the prominent pericyte-endothelial interactions forming the blood-brain barrier (16). Reduction in pericytes abundance, pericyte detachment from capillaries, and accrual of senescence markers are associated with microvascular fragility and dysfunction, as observed in aging, ischemic disease, fibrotic and neurodegenerative disorders, diabetes, tumors, and COVID-19 (17–21). Moreover, capillary pericytes modulate blood flow in different organs. Mice with brain pericyte ablation reportedly developed an acute blood-brain barrier breakdown, severe blood flow decrease, and rapid neuron loss (22). Pericyte contraction participates in the no-reflow phenomenon, the failure of blood to reperfuse an ischemic tissue after the vascular obstruction has been removed or bypassed (23–26).

In addition to their vascular functions, pericytes exert immunomodulatory tasks, sensing inflammatory stimuli through pattern-recognition receptors, and promoting monocyte and neutrophil migration and homing *via* a PDGFRβ signaling mechanism (27, 28). They also express ligands interacting with activated T cell receptors during adaptive immunity responses (29).

Lastly, pericytes play critical roles in tissue restoration, promoting, and stabilizing reparative neovascularization, as reviewed in Navarro et al. (30). Accordingly, regenerative therapies using exogenous pericytes have been preclinically tested (31–37). Pericytes were delivered either as dispersed preparations or as a part of a “tissue-engineered” product to models of myocardial infarction (MI) (33, 36, 37), congenital heart disease (34, 35), limb ischemia (38), muscular dystrophy and skeletal muscle injuries (39–42), blood-brain barrier disorders (43–48), diabetic retinopathy (49–51), and kidney fibrosis (52). These preclinical studies have paved the way for future therapeutic applications in human diseases.

## 5. Antigenic profile of pericytes in relation to function and localization

Like other mesenchymal cells, pericytes are positive for CD105, CD29, CD44, and CD90 and negative for CD45 and CD31. They typically express Platelet-Derived Growth Factor Receptor beta (PDGFRβ), the cell surface glycoprotein MUC18 (CD146), GTPase signaling 5 (RGS5), chondroitin sulfate proteoglycan 4 (NG2), myosin heavy chain 11 (Myh11), and the transmembrane metalloprotease CD13 (12, 53–55).

The expression of antigenic markers can vary according to organ location. Capillary and adventitial pericytes express both NG2 and PDGFRβ. However, capillary pericytes are positive for CD146, whereas adventitial pericytes are positive for CD34 and negative for CD146 (3, 12, 56, 57). Kramann et al. and Crisan et al. illustrated a difference in the antigenic profile of pericytes according to their vascular site. They classified the pericytes into two types. Capillary (type I) pericytes (NG2^+^/Desmin^–^/α-SMA^–^) and pre-capillary (type II) pericytes (NG2^+^/Desmin^+^/α-SMA^+^) (58, 59). Bergers et al. identified post-capillary (type III) pericytes (NG2^–^/αSMA^+^) (60). Moreover, coronary artery pericytes were classified as endocardial-derived (NG2^–^/ PDGFRβ^+^/αSMA^–^) and epicardial-derived (NG2^+^/PDGFRβ^+^/αSMA^+^) (61, 62). Three different subgroups of aortic pericytes were identified based on similarities to fibroblasts (PDGFRβ^+^/PDGFRα^+^/CD34^+^), VSMCs (PDGFRβ^+^/αSMA^+^), and neural cells (PDGFRβ^+^/PLP1^+^/CNP^+^) (63).

Pericytes can change phenotype when transiting from a quiescent to an activated state. NG2 distinguishes topical pericyte properties such as the capacity to control vascular permeability in the brain (64–66), participation in myofibroblast differentiation during the process of cardiac and renal fibrosis (67, 68), and progenitor cell activity in the coronary microvasculature (69). The concurrent expression of CD133, CD34, and Kinase Insert Domain Receptor (KDR) identifies fetal aorta-derived pericytes’ ability to differentiate into endothelial or muscular lineages (15). Adult aortic pericytes express the stemness marker SRY (sex determining region Y)-box 2 (Sox2) (12). and microvascular cardiac pericytes express OCT3/4, GATA−4, and Homeobox protein NANOG (32). The upregulation of another pluripotency gene, Krüppel-like factor 4 (KLF4), marked the switch of perivascular cells to a less differentiated state in tumors (70).

In angiogenesis, the transition from quiescent to actively proliferating pericytes is accompanied by profound changes in the antigenic and secretory profile (71). The expression of alpha-smooth muscle actin (α-SMA) and the downregulation of protein tyrosine kinase Tie2 reportedly mark the pericyte participation in pathological angiogenesis (72–74). Using RNA-Seq transcriptomics, we unveiled new markers distinguishing the differentiation stages of human cardiac pericytes into pro-angiogenic, contractile cells. Naive pericytes express the Cell Adhesion Molecule 3 (CADM3). The expression of the angiogenesis-related Cellular Retinoic Acid–Binding Protein 2 (CRABP2) and Aquaporin 1 (AQP1) distinguished contractile pericytes from naive pericytes and coronary VSMCs (55). In addition, we reported that epigenetic modifications can influence the pericyte pro-angiogenic activity (75). Transplantation of human adventitial pericytes to a murine limb ischemia model improved perfusion recovery and muscular angiogenesis. The therapeutic effect varied among different donor lines. Whole genome screening showed that this variation was significantly associated with the DNA methylation state at the promoter and gene body levels (75).

Gubernator et al. identified two types of skeletal muscle pericytes relevant to adipogenesis and myogenesis (76). Type I pericytes’ antigenic profile (NG2^+^/Nestin^–^/PDGFRα^+^) is linked to a differentiation fate to adipocytes and is associated with fat accumulation (77). Type II expressional profile (NG2^+^/Nestin^+^/PDGFRα^–^) is linked to myocyte commitment and associated with skeletal muscle regeneration (78).

## 6. Distinction from other cells within the perivascular niche

The microenvironment within or in proximity to the blood vessel wall, the perivascular niche, contains mesenchymal stromal cells (MSCs), fibroblasts, and pericytes. Perivascular MSCs express CD146, CD105, alkaline phosphatase (ALP)^+^, Stro-1, and VCAM1 in humans, and CD105, PDGFRα, Sca1, CD44, CD29, and VCAM1 in the mouse (79). It remains unclear whether MSCs and pericytes have a lineage relationship or represent functionally distinct mesenchymal populations that share a perivascular location. Some studies support the possibility that pericytes constitute a more primitive version of MSCs (15, 80), while others suggest that all MSCs are pericytes with some narrow exceptions (81). In line with the latter possibility, a study from Crisan demonstrated that pericytes isolated from different organs express almost all the known markers of MSCs, especially CD10, CD13, CD44, CD73, CD90, and CD105 (82).

Moreover, a strict relationship exists between fibroblasts and pericytes. Fibroblasts are mesenchymal cells in the interstitial space of all organs (83). They express mesenchymal markers such as vimentin, PDGFRα, and type I collagen, along with some tissue-specific markers, such as Transcription Factor 21 (TCF21) and periostin (84). In the brain, perivascular fibroblasts surround cortical penetrating arterioles and their branches in the arteriole-capillary transition zone. Still, they are absent in the capillary zone where pericytes prevail (85). Recent single-cell transcriptomics studies in mice showed that perivascular fibroblast-like cells in the adult mouse brain express unique markers compared with other mural cells (86–88). In the heart, the periostin-expressing myofibroblast originates from tissue-resident fibroblasts and plays critical roles in adaptive healing and fibrosis, according to genetic lineage tracing of cardiac cells (84). A study in the Zebrafish, using a combination of *in vivo* live imaging, genetic ablation, and CRISPR mutant analysis, demonstrated that perivascular fibroblasts play dual roles in stabilizing nascent intersegmental vessel sprouts from the dorsal aorta and later functioning as pericyte progenitors (89). Furthermore, Birbrair et al. distinguished two *bona fide* pericyte subtypes in the skeletal muscle interstitium of mice, naming them type-1 (Nestin-GFP^–^/NG2-DsRed^+^) and type-2 (Nestin-GFP^+^/NG2-DsRed^+^) pericytes. *In vitro*, type-1 pericytes were profibrotic, and type-2 pericytes were myogenic. The latter fate was confirmed after transplantation of type-2 pericytes in young mice and, to a lesser extent, in older mice. Moreover, type-2 pericytes participate in normal and tumoral angiogenesis (77, 78). Therefore, transition states and shared functions between pericytes and perivascular fibroblasts may coexist in disease states.

Given that there is no single-specific pericyte marker, adopting the concept of “Identifying Package’ seems more plausible (82).

## 7. Current protocols for the derivation of pericyte populations

**FIGURE 2:**
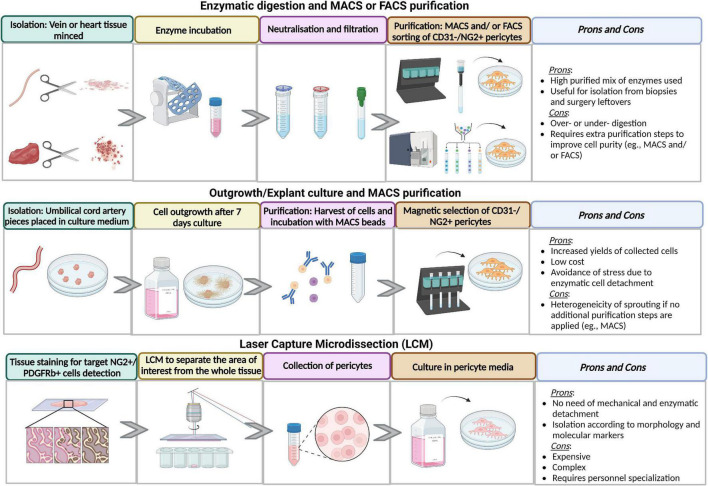
Advantages and disadvantages of different methods of isolation and purification methods. Figure created in Biorender.com source.

The different steps of pericyte derivation and purification are illustrated in Figure 2.

### 7.1. Tissue harvesting and pericyte extraction

The harvesting procedure can affect the subsequent isolation steps. For instance, cardiac surgeons use two techniques to prepare saphenous veins for coronary artery bypass grafting. Removal of perivascular tissue from the vein may damage the adventitia, thereby precluding the isolation of resident pericytes. We prefer to isolate pericytes from veins obtained through the “no-touch” technique (90, 91). The optimal time from harvesting to cell isolation varies according to the tissue source. Blood vessels can be stored for up to 7 days, while cardiac samples must be processed for digestion within 48 h.

The isolation is generally performed using enzymatic digestion or microdissection techniques. The enzymatic digestion approach is particularly useful for pericyte isolation from biopsies and surgery leftovers. The tissue is finely chopped and subjected to one or multiple rounds of enzymatic digestion. Samples with a high collagen content should undergo mechanical dissociation to increase the cell yield (39, 46, 47, 92, 93). Quality control of reagents’ purity and stability includes checking the batch enzymatic activity, determining the activity decay with storage, and ensuring that the final concentration is appropriate to the tissue source and sample quantity. A highly purified mix of Collagenase I and II, commercially available as Liberase TM, is used to digest and isolate pericytes from human and swine saphenous veins and heart (12, 32–35). A mix of Collagenase Dispase, Dnase I, and Tosyl-L-lysyl-chloromethane hydrochloride (TLCK) is used to isolate swine and bovine pericytes from the brain (45). A Papain and Dnase I are employed to isolate mouse brain pericytes (43). A precise tuning of the enzyme volume in relation to the tissue weight is essential to ensure efficient cell isolation (3, 12). An excessively aggressive procedure can impact cell viability, while incomplete tissue processing can compromise cell yield. The incubation time for tissue digestion is also critical. For pericytes extraction from the heart, we treat the chopped tissue with Liberase for up to 1 h at 37°C under gentle rotation.

Microdissection is usually applied to outgrowth/explant cultures. The tissue is dissected into small pieces using a scalpel or scissors, washed to avoid red blood cell contamination, and distributed over the surface of a culture dish with or without extracellular matrix (ECM) coating. Pre-warmed media is then added to cover the fragments completely. Explanted cultures are kept undisturbed for a few days until pericytes become visible as migrated cells from the tissue pieces (31, 41, 94). The advantages of this isolation technique are the increased yield of collected cells, low cost, and avoidance of the destructive stress caused by enzymes (95). However, enzymatic pre-treatment may sometimes become necessary, as in the case of micro-vessels from the placenta (91).

Laser capture microdissection (LCM) enables the operator to separate the cells of interest from the whole tissue specimen under the microscope using a cutting beam of UV laser (96, 97). This separation strategy does not require prior mechanical or enzymatic digestion. Thus, it allows for preserving the native cell phenotype, gene expression, and function (98). The disadvantage is represented by costs, complexity, and personnel specialization (99, 100). LCM was used to isolate pericytes from snap-frozen human and mouse brains (101).

Enzymatic dissociation and microdissection are generally followed by separation of antigenically defined cell populations by FACS or immunomagnetic columns. This could be associated with using selective culture media during cell expansion (31, 102).

### 7.2. Purification

Table 1 shows the negative and positive markers used for sorting, while Table 2 illustrates the methods for enhancing purity.

**TABLE 1:** Positive and negative pericytes’ markers in different species and tissues.

Organ	Species	Positive markers	Negative markers	References
Brain	Mouse	CD13, PDGFRβ, NG2, α-SMA	CD31, CD45, CD11b, GFAP, GLAST-1, GFAP, AQP4, MAP2, NeuN, VWF, Iba1	(44, 166–169)
	Swine	NG2, α-SMA	GFAP, VWF	(45)
	Human	NG2, PDGFRβ	IB4	(170)
Skeletal muscle	Mouse	NG2, PDGFRβ, CD146, Nestin	Pax7, Myf5, FSP1, Scleraxis, CD31, CD45	(39, 77, 78, 171)
	Swine	NG2, PDGFRβ, α-SMA	Not examined	(40)
	Human	CD146, PDGFRβ, NG2, SMA and ALP	CD34, CD45 CD31, CD62L, CD71, CD106, CD117 CD133	(111, 172)
Heart	Mouse	NG2, PDGFRβ, CD146	CD31, CD34, CD45	(173)
	Human	CD34, NG2, PDGFRβ, Vimentin	CD31, CD146	(55)
	Swine	CD34, NG2, PDGFRβ, α-SMA	CD31, CD146, CD45	(34, 35)
Aorta	Mouse	NG2, PDGFRβ, α-SMA	CD31, VE-Cad	(64, 102)
Lung	Human	3G5, PDGFRβ, α-SMA, CD146	Wnt5a, CD31, CD45, CD34	(174, 175)
	Mouse	PDGFRβ, NG2, CD146, α-SMA	CD31, CD45, CD326, Ter199, EpCAM	(176–178)
Retina	Mouse	PDGFRβ	CD31	(179)
	Rat	NG2, PDGFRβ, α-SMA. Desmin	vWF, GS, GFAP, SMMHC	(49)
	Bovine	PDGFRβ, NG2	VWF, Collagen IV	(50, 180)
	Human	PDGFRβ	CD31	(179)
Bone marrow	Human	CD146, α-SMA	CD45, CD34, CD31	(105)
Adipose tissue	Human	CD146	CD31, CD45, CD56, CD34	(181)
	Equine	CD146, CD34	CD45, CD144	(42)
Placenta	Human	CD146, NG2, PDGFRβ, α-SMA, Desmin	CD31, VWF	(181, 182)
Endometrium	Equine	NG2, CD146	CD34, CD45, MHC-II	(183)
Umbilical cord	Human	CD146	CD34, CD45, CD31	(31, 172)
Saphenous vein	Swine	CD34, NG2, PDGFRβ	CD31	(33)
	Human	CD34, NG2, PDGFRβ	CD31	(12)
Kidney	Mouse	PDGFRβ, Coll1a1, NG2	CD31, CD45, CD326, CD144, CD11b	(178, 184)
Liver	Human	PDGFRβ, α-SMA, CD146	CD34	(174)
Intestine	Mouse	ALP, NG2, PDGFRβ	CD31	(100)
Ear (cochlea)	Mouse	NG2, PDGFRβ, Desmin	VWF, GS-IB4	(185)
Somatic cells from human body: iPSC-PCs	Human	PDGFRβ, NG2 CD13, CD146	CD31, CD144	(144–148)

**TABLE 2:** Different strategies and methods for enhancing purity in isolation and purification of pericytes from different species and tissues.

Methods	Organs	Species	Purity enhancement	References
Enzymatic digestion (isolation)	Retina	Bovine, human, rat	- Selective media (DMEM, BCS)	(49, 50, 138, 139, 180)
	umbilical cord	Human	CD146-MACS	(137)
	Brain	Human, mouse, swine	- Selective media (DMEM)	(43, 45, 92, 122, 166)
	Kidney	Human	CD146-FACS	(128, 129)
	Lung	Human	- PDGFRβ-MACS - Selective media (anti-Trop2)	(109, 130, 131)
	Endometrium	Equine	Mucin-Dynabeads	(183)
	Skeletal muscle	Swine	- Selective media (DMEM).	(40)
MACS (isolation and purification)	Heart	Human, swine, Mouse	CD34-MACS, 3G5-MACS	(35, 55, 94)
	Saphenous vein	Human, Swine	CD34-MACS	(12, 33)
	Placenta	Human	CD146-MACS	(31)
	Liver	Human	CD146, PDGFRβ-FACS	(174)
	Bone marrow	Human	CD146-MACS	(105)
FACS (isolation and purification)	Brain	Human, mouse	CD90-FACS, CD13-FACS	(44)
	Skeletal Muscle	Mouse	PDGFRβ-FACS, CD146-FACS	(38, 39)
	Retina	Mouse	PDGFRβ-FACS	(51)
	Adipose Tissue	Equine	CD146-FACS, CD34-FACS	(42)
	Kidney	Mouse	NG2-FACS	(52)
	Skin	Human	VLA-1 -FACS	(112, 135)
	Lung	Mouse	PDGFRβ-FACS	(177)
Laser microdissection (selective isolation)	Brain	Human, mouse	Isolation of vessels < 10 μm only	(101)
Explant outgrowth (isolation)	Umbilical cord	Human	NG2-MACS	(31)
	Skeletal muscle	Human, mouse	- Repeated re-culture. - ALP-FACS	(41, 111)
	Brain	Human	Selective media (DMEM)	(120)
	Small intestine	Mouse	Selective media (megacell enriched)	(100)
	Ear (cochlea)	Mouse	Selective media	(185)
	Aorta	Mouse	Selective media	(102)

In preparation for immunomagnetic sorting, the crude preparation from digestion or microdissection is passed through 70-, 40-, and 30-μm strainers to eliminate debris. Using immunomagnetic columns, cells are labeled with magnetic microbeads conjugated with antibodies and exposed to a magnetic field (90). Three platforms are available for this purpose: the magnetic separator, a column-free magnetic separation fitting tube containing cells labeled with antibodies, and magnetic particles. The labeled cells are retained in the tube walls, and the unlabeled cells are discarded. *MACS columns* and *Dynabeads* are widely used to isolate pericytes due to the simplicity and rapidity of the procedure (32–35, 94, 103). The harsh magnetic field may cause damage or rupture of the cell membranes of fragile cells. Furthermore, column-based separations generally employ a limited number of positive and negative markers, resulting in a semi-purified cell population.

Fluorescent-Activated Cells Sorting (FACS) is more reliable than immunomagnetic columns. The final population is highly pure for the selected antigens, but cell yields may be insufficient for immediate applications requiring large cell quantities. The strategy for gating cells can make a profound difference in the outcomes (12, 39, 41, 42, 44, 52, 90, 104, 105).

### 7.3. Expansion

This step requires fine-tuning environmental conditions for pericytes’ rapid and consistent growth and suppression of contaminant cells. Essential elements and components include appropriate cell seeding density, flasks/plates coating, media, growth factors (GFs), and antibiotics.

Pericytes have elongated cytoplasmic processes which enable the *in vitro* cell-cell communication necessary for the production and secretion of their “sensome,” a novel term used to indicate different GFs sensed by these cells (106). Hence, calculating the correct cell seeding density is essential to avoid growth arrest and senescence caused by the lack of physical contact. To support pericytes adherence to the culture surface, 0.1–0.2% (w/v) gelatin solution is widely used for precoating.

Dulbecco’s Modified Eagle Medium (DMEM) has been used for the expansion of pericytes sourced from the brain (107), kidney (108), lung (109), skeletal muscle (110, 111), and skin (112). A DMEM version including many additional factors (e.g., zinc, thymidine) could help improve the growth rate (113). The addition of Fetal Bovine Serum (FBS) is debated due to the reagent’s complexity (114–116). Additionally, pericytes from the heart (32), saphenous vein (117), and umbilical cord (31) were expanded in Endothelial Cell Growth Medium 2 (EGM-2), while bone marrow pericytes were cultured with α-MEM containing FBS (105). Antibiotic treatment with penicillin and streptomycin is usually added to the culture medium to reduce contamination.

The expansion by single-cell cloning allows for enriching progenitor cells within the bulk preparation of sorted pericytes (118, 119). Single cells are sorted into a Terasaki multi-well plate to give rise to clones and subclones (12, 111). Mechanical and chemical stress during the sorting procedure can result in poor recovery and reduced clonogenic expansion. Moreover, the FACS system requires a high level of daily maintenance and sterility to preserve the cell culture environment from possible contamination.

Even adopting a very stringent expansion protocol, expansion efficiency may vary depending on the donor’s tissue source, age, and clinical conditions. Using a good manufacturing practices-compliant SOP, we expanded dozens of pericyte lines from the saphenous vein and heart. Neonatal cardiac pericytes grew faster and better than adult pericytes. From a sample weighing less than 100 mg, we could obtain ≈20 million cells at passage 5, in 4–6 weeks of culture expansion. Ten times larger amounts of the vein were necessary to reach these quantities (32). Moreover, older patients’ pericytes undergo proliferative senescence at earlier passages (75). Experts generally consider a cell preparation’s optimum purity and viability above 90%.

## 8. Isolation of pericytes from human tissues

Pericytes have been isolated from different tissues.

### 8.1. Brain

Brain pericytes could be isolated from the white and the gray matter using enzymatic digestion and explant culture (92, 107, 120–122). They express PDGFRβ, αSMA, CD13, NG2, CD146, and desmin (121). Brain pericytes are expanded in culture with DMEM/F12 supplemented with 10% (v/v) FBS (120).

### 8.2. Heart

Cardiac pericytes were isolated from atria and ventricles using enzymatic digestion, filtration, and immunomagnetic separation to obtain CD31^–^/CD34^+^ cells (21, 32, 55). Cardiac pericytes are expanded in a dedicated medium supplemented with human recombinant GFs (ECGM2) and 2% FBS. This medium was superior to mesenchymal stromal cell media such as MACS + 10%FBS and DMEM + 20%FBS (32).

### 8.3. Skeletal muscle

Skeletal muscle-derived pericytes were mainly isolated by explant culture. Distinct populations, such as alkaline phosphatase/CD56^–^ cells and CD146^+^/NG2^+^/αSMA^+^/ CD45^–^/CD31^–^/CD34^–^ cells, could be purified by FACS or immunomagnetic microbeads (77, 78, 93, 123, 124). Type I collagen-coated plates are expanded in DMEM containing 5% (v/v) FBS (41, 111).

### 8.4. Bone marrow

Bone marrow pericytes can be isolated immunomagnetically using a microbead cell sorting strategy. Bone marrow samples are treated with Ficoll Histopaque 1,077 to obtain mononuclear cells. Firstly, cell populations are negatively purified from CD45^+^/CD34^+^ fractions. Then, CD146^+^ cells are selected and further expanded, with final confirmation of purity for the CD45^–/^CD34^–^/CD146^+^ combination by flow cytometry and immunocytochemistry (105). The bone marrow pericytes can be grown in α-MEM basal medium supplemented with 20% FBS (105, 125).

### 8.5. Kidney

Kidney pericytes are essential in regulating permeability, formation of myofibroblasts, angiogenesis, and renal medullary blood flow (126–128). After tissue digestion using a collagenase cocktail (IA, II, and IV), cells are FACSorted to obtain a population of CD146^+^/CD34^–^/CD45^–^/CD56^–^ pericytes, which also express the classical makers PDGFRβ and NG2. Culture expansion is performed on gelatin-coated dishes (108, 128, 129).

### 8.6. Lung

The methods for human lung pericyte isolation include enzymatic digestion, magnetic microbead sorting, and FACS selection (109, 130–132). Then, cells are sorted to collect the CD45^–^/CD31^–^/CD326^–^/PDGFRβ^+^ fraction (109, 130). In addition, Kramann et al. isolated lung pericytes using a selection medium that contained AY16 (an anti-Trop2 monoclonal antibody)-DT3C conjugate to eliminate epithelial stem cells and endothelial cells (131). The modified medium selected cells express PDGFRβ and chondroitin sulfate proteoglycan 4 (CSPG4).

### 8.7. Skin

Pericytes have been isolated from the neonatal foreskin or adult female breast skin (133). After digestion with type II collagenase or dispase (134), a VLA-1^bri^/CD45^–^ population was sorted by FACS or immunomagnetic microbeads (112, 133, 135). In addition, skin pericytes express Human high molecular weight-melanoma-associated antigen (HMW-MAA), PDGFRβ, desmin, αSMA, α1β1-integrin, α- and γ-actin isoforms, and myosin (136).

### 8.8. Umbilical cord

Human umbilical cord pericytes can be isolated by enzymatic digestion and explant culture (31). Helmbold et al. performed immunomagnetic sorting of cells using CD146-conjugated microbeads to separate the CD146^+^ population (137). Cathery et al. isolated pericytes by explant culture. The migrated cells underwent immunomagnetic sorting, and the CD31^–/^NG2^+^ population was acquired (31). All the isolated populations showed the characteristic negative expression of CD34, and positive expression of CD146 and NG2.

### 8.9. Retina

After separation from the pigmented layer, the human retinal pericytes are isolated by enzymatic dissociation. The cells are seeded on gelatin-precoated dishes and cultured with DMEM/F-12 containing FBS. The characteristic morphology of retinal pericytes is identified by immunofluorescent staining of NG2, αSMA, glial fibrillary acidic protein, and cytoskeletal structural proteins (138, 139).

## 9. Isolation and expansion of pericytes from animal tissues

Although human cells constitute the final therapeutic product for patients, animal-derived cells represent a suitable surrogate in preclinical safety and efficacy studies (140). Using donors and recipients from the same species, ideally from the same litter, eliminates the need for immunosuppression, which is instead required when transplanting human cells into an animal model (33). Recent technological progress makes swine-derived cells, tissues, and organs attractive for immune-compatible xenotransplantation. The cells are genetically modified to remove the immunogenic factors. Human transgenes can be expressed to suppress rejection (141).

Lack of uniformity in cell manufacturing between human and animal species might influence the interpretation and translatability of preclinical results. Therefore, robust approaches for isolation, culture, expansion, characterization, and appropriate *in vivo* testing of pericytes in larger animal species are of utmost importance.

### 9.1. Protocols for isolation and expansion

Animal primary cells may incur bacterial or fungal contamination because of the poor sterile conditions used to collect the starting material. Therefore, using antibiotics and Amphotericin B and manipulating cells in the laminar flow cabinets is fundamental to maintaining sterile conditions and avoiding the spread of cross-infection to the cells. An accurate selection of the proper culture medium, GFs, percentage of serum, and plate coating is key to simulating the *in vivo* environment and allowing adequate expansion (142). Significant changes may need to be introduced to adapt protocols initially devised for human pericytes. For instance, swine pericytes grown in autologous or allogeneic serum displayed a 100% success of expansion and high doubling time compared with the standard xenogeneic FBS employed for the growth of human pericytes (12, 32, 33).

## 10. Pericyte generation from iPSCs

Induced Pluripotent Stem Cells (iPSC) derived through reprogramming somatic cells with pluripotency factors (OCT3/4, SOX2, c-MYC, KLF4) can be differentiated to a specific cell line, including pericytes. 3D-culture systems of iPSC-derived cardiovascular cells have provided a novel *in vitro* model to study the interplay among different cell types and potentially develop new personalized treatments for congenital and acquired cardiac diseases (116, 143–145).

The developmental origin of cardiac pericytes is thought to be the epicardium. Differentiation of iPSCs to epicardial cells can be achieved through the modulation of Wnt/β-catenin signaling and treatment with pericyte growth factors, such as PDGF-BB (144, 146, 147). Szepes et al. applied a mesodermal induction using BMP4, VEGFA, and activation of Wnt/β-catenin signaling with further VEGFA and TGFβ inhibition. FACS staining and sorting for PDGFRβ and CD31 markers and further expansion were carried out for antigenic characterization, gene expression, and function in an endothelial tube formation assay. A tri-cultured bioartificial cardiac tissue model was generated using iPSC-derived pericytes, cardiomyocytes, and ECs to study vascularization and fibrosis *in vitro* (145). Faal et al. reported two protocols using mesoderm and neural crest to obtain brain pericytes from iPSCs generated from healthy subjects and Alzheimer’s disease patients. Briefly, human iPSCs were cultured on a Matrigel-coated plate in a mesodermal induction medium with growth factors. The activation of Wnt/β-catenin signaling initiated neural crest differentiation through specific medium and supplements. Mesodermal and neural crest-derived cells were differentiated in pericytes seeded on gelatin or Matrigel-coated plate. Both pericyte types were passaged, split, and kept in the pericyte medium for their characterization and functional trans-endothelial electrical resistance assay with ECs (146). Jamieson et al. generated pericytes from human iPSCs obtained from mesodermal lineage. In brief, iPSCs were cultured on a feeder layer of mouse embryonic fibroblasts and gelatin-coated plates. For the differentiation, iPSCs were detached with EDTA, strained, and seeded on a collagen-coated plate using α-MEM, FBS, and β-mercaptoethanol. Following further detachment, they were grown in EC growth medium, TGFβ inhibitor, and VEGF to promote vascular cell specification and proliferation. After an enzymatic dissociation, DMEM + FBS was added to an uncoated plate to support the pericyte selection. Generated iPSC-derived pericytes were used for developing a microvessel model of the blood-brain barrier (148).

## 11. Standardization and harmonization

It has been shown that fewer than one-third of biomedical papers can be reproduced, resulting in associated costs of about $28 billion each in the US only, mainly attributable to the wasted follow-on work that the initial study has inspired (149, 150). Reproducibility of results from peer-reviewed papers by researchers at pharmaceutical companies failed at rates upwards of 75% (151). This reflects the inconsistent use and communication of standardized processes to achieve efficiency, quality, and uniformity of performance. The main body of information on pericytes methodologies can be retrieved from original papers, which show large variability regarding the depth of details. The following paragraphs are devoted to suggesting improvements to current methodologies.

### 11.1. Increasing reproducibility

It is highly recommended that the entire SOP is published as an accompanying material or companion paper and deposited in an accessible database (152). Open access public repositories, such as Zenodo,^1^ SEEK (153), OpenAIRE,^2^ FAIRsharing^3^ are available for SOP submission.

We have previously reported the SOP for GMP production of human adventitial pericytes, which consists of: (i) Cell Isolation, (ii) Cell expansion (iii) Cell splitting, (iv) Cell freezing/thawing, and (v) Extraction of serum from blood. Each section contains a checklist of 6 to 44 operational steps that the named operator has to do (154). An attached document reports details of the collection, the code associates the sample to the NHS patient’s records and informed consent form and ethical approval, code and storage conditions for reagents, equipment, and deviations from the protocol. An electronic copy was stored in a dedicated folder of the institutional server at the University of Bristol.

### 11.2. Upgrading

GMP manufacture is a quality assurance system pharmaceutical companies use to ensure that the final medicinal product meets proper specifications. GMP covers both the manufacturing and testing of the final product. Translation of the GMP concept to cell therapy and tissue engineering remains vague, mainly because most cell products are early stage prototypes studied in academic laboratories (155). To the best of our knowledge, our previous study remains the only one to have upgraded human adventitial pericytes to GMP (154). We have verified that the clinical-grade, xeno-free reagents worked like the research-grade ones. Then, the whole production process was transferred to the GMP facilities at the NHS Blood and Transplant (NHSBT) Unit in Bristol. NHSBT is authorized by the Human Tissue Authority (HTA) and Medicine and Healthcare products Regulatory Agency (MHRA) to manufacture advanced therapy products for cell therapy trials.

### 11.3. Quality control

Introducing quality and quantity control (QQc) systems to produce pericytes provides two significant improvements. Namely, it reduces production costs and certifies the cell product. We process only samples that exceed a minimum weight. Using this quantity control criterion, we calculated a 33% cost saving thanks to avoiding expansion failure.

Quality and quantity control analyses include a cell viability/death test by Calcein/EDthIII assay and Trypan Blue, repeated antigenic characterization using both positive and negative markers to exclude that contaminant cells had emerged during expansion, karyotyping to ensure that cells do not accumulate chromosomic damage and functional characterization (33, 75). Among the most widely used tests, the Matrigel assay demonstrates the pericytes’ capacity to improve the network-forming activity of ECs, while measuring angiogenic factors in the media allows recognizing the pericytes’ ability to release typical angiogenic factors such as Hepatocyte Growth Factor (HGF) and Angiopoietin 2 (Ang-2) (32). More sophisticated tests aiding the characterization of pericytes include the assessment of contraction and relaxation using the Electrical Cell Intuitive Sensing (ECIS) platform, barrier integrity by analyzing membrane current, permeability using co-culture of ECs and pericytes on Transwell filters and microfluidic models, mechano-transduction properties, and circadian rhythms regulating the crosstalk between pericytes and endothelial cells (32, 55, 156–159).

### 11.4. Integrating methodologies

All the derivation techniques described above have pros and cons. Appropriate combinations could increase the purity and yield of production. For instance, repeating immunomagnetic separation can help maintain a high purity level during expansion. When massive production of pericytes is needed, using Multi-Flasks provides an optimal cell culture environment, simplifies the workflow by eliminating multiple steps, and reduces the risk of contamination. A step forward, automated pericyte culture systems could deliver high throughput, high purity, and high yield cell growth, perform large-scale screening, and run several experiments in parallel, thereby allowing for reducing inter-assay variability.

Another attractive approach is based on the use of multi-omics. Potent technologies such as single-cell RNAseq have been applied to pericyte research, providing a spatial atlas of the vascular niche in different organs, and revealing organ-specific pericyte markers and identities (88, 160, 161). In the brain, a continuum phenotypic change (zonation) along the arteriovenous axis for ECs was contraposed by a punctuated continuum for mural cells. Among the latter population, pericytes coexisted with a population of perivascular fibroblast-like cells localized at all vessel types except capillaries (88). In the heart, large-scale single-cell and single-nucleus transcriptome analyses identified 6 anatomical regions (162). The vascular compartment included 17 distinct populations of ECs, VSMC, pericytes, and mesothelial cells. Pericytes formed 4 clusters, of which one was transcriptionally distant from other vascular cells, while another constituted a transitional state between pericytes and ECs. The relevance of this new sub-classification should be validated at the level of the functionome. Multifunctional microwell arrays that enable single-cell functional analysis have already been used to study lymphocytes (163). Live-cell imaging and analysis unveiled cell transition dynamics that could not be captured by classical snapshot imaging (164). Other emerging single-cell methodologies promise to define cell heterogeneity on the protein and DNA epigenetics levels (165).

## 12. Conclusion

This review highlighted the significant efforts made by different teams to develop effective protocols of isolation and *in vitro* expansion of human pericytes. A consensus on these protocols and the integration of methodologies will help speed up research at the highest level of reproducibility and translatability. Moreover, additional research is needed to dissect the functional diversification of pericytes according to their zonal distribution and the surrounding microenvironment. More work is also necessary to trace pericytes in tissues during vascular remodeling. Expanding and refining the atlas of different perivascular niches at the single-cell level will clarify the enigmatic phenotype of pericytes and the relevance of expressional deviations in response to organ injury. This novel technological approach should be integrated into the dimensions of space and time, for instance, by clustering dynamic gene expression profiles from single-cell RNA-Sequencing databases and associating transcripts to functionomics analysis. Most studies on pericytes have been performed in rodent models. More research in large animal models is needed to demonstrate therapeutic utility.

## Data availability statement

The original contributions presented in this study are included in the article/supplementary material, further inquiries can be directed to the corresponding author.

## Author contributions

PM was responsible for the final writing and the data presented. All authors conceived and designed the article, read, and approved the final manuscript.

## Footnotes

1
https://zenodo.org

2
https://explore.openaire.eu/participate/deposit-publications

3
https://fair-dom.org/platform/
